# Distinct Modes of Quantum Information Transfer in Power–Law Long-Range Spin Networks

**DOI:** 10.3390/e28101101

**Published:** 2026-10-10

**Authors:** Ellis E. Marshall, Chad C. Nelmes, Tony J. G. Apollaro, Timothy P. Spiller, Irene D’Amico

**Affiliations:** 1 School of Physics, Engineering and Technology, University of York, York YO10 5DD, UK; 2 Sussex Centre for Quantum Technologies, University of Sussex, Brighton BN1 9RH, UK; 3 York Centre for Quantum Technologies, University of York, York YO10 5DD, UK; 4 Department of Physics, University of Malta, MSD 2080 Msida, Malta

**Keywords:** spin chains, quantum information processing, evolutionary computation, quantum state transfer, long-range models

## Abstract

We identify different regimes of quantum state transfer in long-range coupled spin-$\frac{1}{2}$ systems, where naturally occurring power–law interactions enable rapid, high-fidelity transfer with minimal engineering. Across a broad range of interaction profiles, from effectively nearest-neighbour coupling to Coulomb interactions, we show how long-range connectivity fundamentally reshapes the mechanisms underlying information propagation within such systems. For effectively short-range interactions, transfer follows familiar ballistic transfer dynamics: an initially localised excitation spreads across many eigenmodes concentrated within the approximately linear region of the spectrum, enabling robust wavepacket motion. In contrast, increasing long-distance interactions via lowering the power–law exponent α (α=1−2) drives a striking transformation, where the initial state becomes confined to progressively fewer eigenmodes, ultimately reducing the dynamics to the coherent participation of only a few states corresponding to the highest eigenenergies. This spectral localisation gives rise to emergent long-range oscillations between distant sites, revealing a distinct—and faster—transfer mechanism arising from the intrinsic structure of long-range quantum interactions rather than from full-system engineering pathways.

## 1. Introduction

Following the seminal proposal of quantum-advantaged information transfer through unmodulated (Heisenberg) spin chains by Sougato Bose [1], and the introduction of perfect state transfer (PST) via engineered mirror–symmetric systems [2,3], significant effort has been devoted to improving both the speed and robustness of quantum communication via solid-state hardware. Along with a plethora of other quantum information processing tasks through the natural dynamics of spin chains [4,5,6,7,8,9], quantum state transfer holds particular applicability to the scaling of quantum computing hardware. PST protocols enable exact state mirroring in fully engineered spin networks [2,10,11,12,13,14], while ballistic transfer schemes [15,16,17] enable a high-fidelity/quasiperfect state transfer (QPST) that is at times comparably faster than times required for total state mirroring in exclusively nearest-neighbour (NN) settings [18]. These ballistic approaches exploit an encoding of quantum information as a wavepacket in *k*-space [19]. They typically require only boundary-coupling engineering and local magnetic-field control to position the wavepacket within the linear region of the energy spectrum. Previous studies have shown that a linear energy spectrum enables the fastest perfect quantum state transfer [2,3,5,18,20]. In addition to ballistic wavepacket propagation, minimalist engineering approaches that tune none or at most a few couplings or on-site energies can give rise to few-mode coherent oscillations between the end sites [8,21,22,23], enabling QPST, although this generally comes at the cost of very long transfer times.

Despite the natural emergence of power–law long-range (LR) interactions in promising hardware platforms for fault-tolerant universal quantum computation [24,25,26], their potential as quantum buses has received comparatively little attention over the past two decades, with only a limited number of studies to date [12,27,28,29,30,31,32,33]. While extensive research has explored state-transfer protocols in nearest-neighbour systems for high-fidelity transfer [1,2,6,8,9,15,16,20,22,34,35,36,37,38], and, more recently, in next-nearest-neighbour (NNN) extensions [13,17], long-range interacting models have historically received comparatively little attention. The NN and NNN approaches span fully engineered networks and minimally controlled schemes based on boundary-parameter tuning [15,16,17,22,34,39], both of which can achieve PST and/or QPST in XX spin chains. However, these techniques have not yet been systematically extended to genuinely long-range interacting systems, particularly in experimentally accessible system sizes of $N∼10^{2}$ sites [40,41].

In the present work, we focus on the physically relevant regime of experimentally realistic interactions and system sizes, aiming to identify transfer protocols that remain both scalable and operationally viable within feasible coherence times. We focus on a range of values of the decay exponent α defining the power–law interaction, namely α∈[1,10]. These values naturally arise, or can be effectively approximated, across a variety of quantum platforms [42,43,44].

## 2. Spin Model

We introduce the spin-$\frac{1}{2}$, so-called XX (also XY isotropic) model

(1) $${\hat{H}}_{\mathrm{XX}}=\frac{1}{2}\sum_{i<j}J_{i,j}\left({\hat{\sigma }}_{i}^{x}{\hat{\sigma }}_{j}^{x}+{\hat{\sigma }}_{i}^{y}{\hat{\sigma }}_{j}^{y}\right)+\frac{1}{2}\sum_{i}B_{i}{\hat{\sigma }}_{i}^{z}$$

where

(2) $$J_{i,j}=\frac{J_{0}}{r_{i,j}^{\alpha }}$$

are the site-dependent couplings defined by the dimensionless relative positions $r_{i,j}=\left|i-j\right|$ between sites, $J_{0}$ is the maximal bulk coupling energy, and $B_{i}$ are the site-specific magnetic fields/on-site energies. Clearly, as we increase α within Equation (2), it can be seen that Equation (1) tends towards nearest-neighbour (NN) interactions, and as we decrease it, we tend towards full network connectivity. The power–law exponents that commonly arise in quantum systems are α=1 for Coulomb-mediated trapped-ion spin models [45,46], α=3 for magnetic or electric dipole–dipole interactions [42,44,47], and α=6 for van der Waals interactions [48,49]. In this study, we consider values of α from 1 to 10 to elucidate how different effective interaction ranges influence the state-transfer dynamics. As an example, trapped-ion platforms allow the power–law exponent to be tuned experimentally [42,50].

## 3. Quantum State Transfer

The quantum state-transfer (QST) scheme considered here was originally introduced in Ref. [1] and consists of encoding an arbitrary single-qubit state $|\psi_{s}\rangle =\cos\frac{\vartheta }{2}|0\rangle +\sin\frac{\vartheta }{2}e^{i\phi }|1\rangle$ at a designated sender site. The state is subsequently transferred through the natural unitary dynamics of the spin chain and recovered at a different location, called the receiver site, after a time τ. Throughout this work, the sender and receiver are taken to be the opposite ends of the chain, namely sites 1 and *N*, respectively. Let $|\Psi \left(t\right)\rangle =e^{-i{\hat{H}}_{\mathrm{XX}}t}|\Psi \left(0\right)\rangle$ denote the state of the entire spin chain at time *t*. The reduced density matrix of the receiver spin is ${\hat{\rho }}_{r}\left(t\right)={\mathrm{Tr}}_{1,\ldots ,N-1}\left[|\Psi (t)\rangle \langle \Psi (t)|\right].$ The performance of the QST protocol is quantified via the fidelity between the initial sender state and the state reconstructed at the receiver site, averaged over all pure input states

(3) $$\begin{array}{c} \langle F(t)\rangle =\frac{1}{4\pi }\int d\Omega \langle \psi_{s}|{\hat{\rho }}_{r}\left(t\right)|\psi_{s}\rangle \;. \end{array}$$

**Figure 1:**
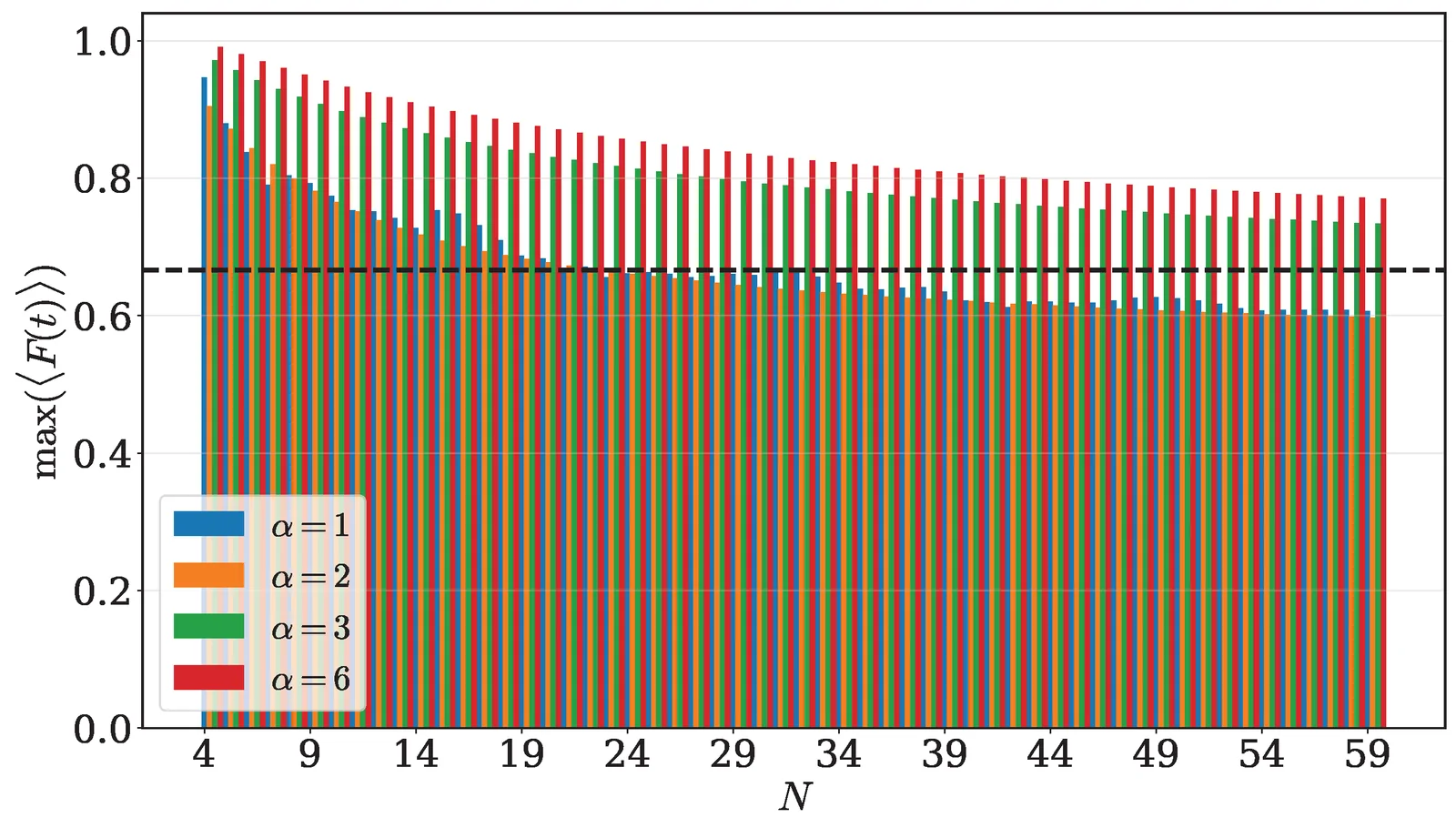
Maximal average fidelity as a function of total number of sites N∈[4,60] for α-chains, evaluated over a total time window $t\cdot J_{0}=N\pi /2$, corresponding to twice the speed limit for perfect state transfer (PST) in nearest-neighbour systems [18]. Horizontal (dashed) line corresponds to the 2/3 limit in which there is quantum advantage over classical means of single-qubit communication [51]. In the large-α limit, the XX (effective) nearest-neighbour spin chain is known to lose its quantum advantage for communication at N≈240 [52].

Initialising all but the sender spin in the fully polarised state, i.e., $|\Psi (0)\rangle ={|\psi_{s}\rangle }_{1}{⊗}_{i=2}^{N}{|0\rangle }_{i}$, we can exploit the model’s U(1)-symmetry to restrict the dynamics to the single-particle preserving subspace, yielding from Equation (3) [1]

(4) $$\begin{array}{c} \langle F(t)\rangle =\frac{1}{2}+\frac{\left|f_{1}^{N}\left(t\right)\right|}{3}+\frac{{\left|f_{1}^{N}\left(t\right)\right|}^{2}}{6}\;, \end{array}$$

where $f_{1}^{N}\left(t\right)=\langle N|e^{-i{\hat{H}}_{\mathrm{XX}}t}|1\rangle$ is the single-particle transition amplitude from site 1 to site *N*, adopting the notation $|i\rangle =|0_{1}\rangle ⊗\cdots ⊗|1_{i}\rangle ⊗\cdots ⊗|0_{N}\rangle$, with i∈{1,…,N}. Hence, the average fidelity 〈F(t)〉 is a monotonically increasing function of the absolute value of the transition amplitude, which is given by

(5) $$\begin{array}{c} f_{1}^{N}\left(t\right)=\sum_{k=1}^{N}v_{1k}v_{Nk}^{*}e^{-i\omega_{k}t}\;, \end{array}$$

where $\left\{\omega_{k},|v_{k}\rangle \right\}$, with $|v_{k}\rangle =|v_{1k},v_{2k},\cdots ,v_{Nk}\rangle$, are the eigenvalues and eigenvectors of ${\hat{H}}_{1}$, the Hamiltonian in the single-particle sector. Clearly, when Equation (3) equals unity at some time τ where the fidelity is maximal, this signifies PST. Any spin-chain configuration that yields an average fidelity value at τ less than unity but still ≳0.99 is classified as QPST. Figure 1 shows how the state-transfer fidelity of completely unmodulated long-range spin chains with power–law interactions, described by Equation (1), deteriorates with increasing system size *N* for different values of the interaction exponent α. This demonstrates that some sort of (preferably minimal) intervention or modulation is required, to deliver QPST from such chains as *N* is increased. This is the motivation for the work presented here.

## 4. Optimisation Approach

We employ evolutionary computation methods for this investigation. In particular, genetic algorithms (GAs) [53,54,55,56,57] operate by initialising a population of candidate solutions (“individuals”), each encoded by a set of parameters that determine their performance with respect to a given objective. This population is then evaluated through a fitness (or cost) function that quantifies how well each individual performs within a specific environment. Individuals with higher fitness are preferentially selected to generate offspring through genetic operators such as crossover and mutation, thereby exchanging and modifying information across candidate solutions. The resulting offspring constitute a new generation and are reintroduced into the same environment for further evaluation. This iterative process is repeated over successive generations, progressively refining the population toward higher fitness. Termination is typically determined either by convergence when no significant improvement in the best fitness is observed or upon reaching a predefined maximum number of generations.

**Figure 2:**
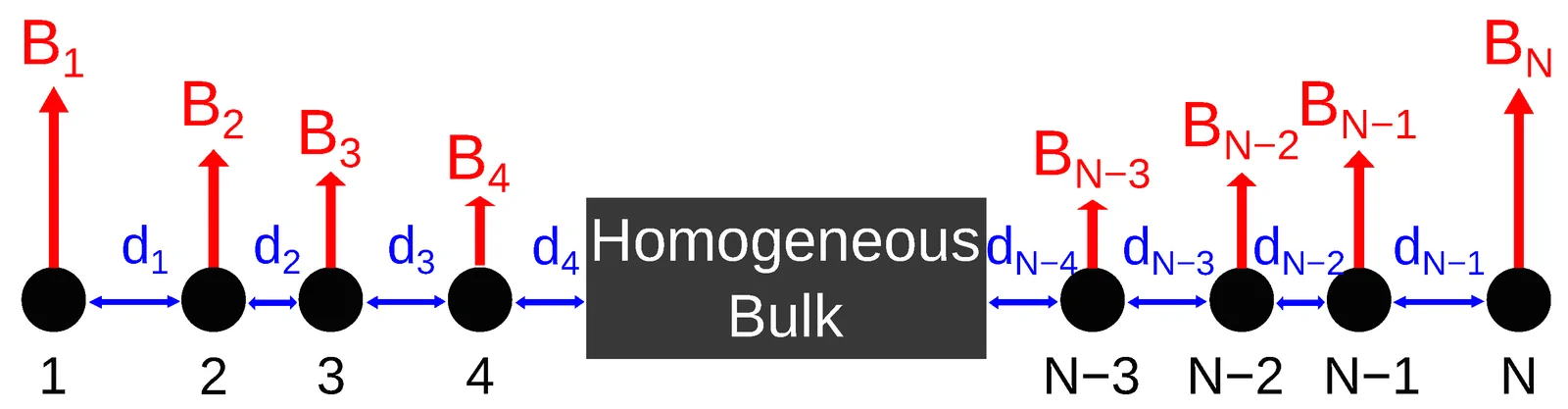
A diagram showing an example chain of length N with four optimised site positions, with $d_{i}$ the distance between two adjacent sites and on-site energies, $B_{i}$, on each end of the chain, preserving mirror symmetry.

GAs have been successfully employed to identify novel Hamiltonian configurations for the optimisation of information processing tasks [17,37,58,59]. In fact, recent studies suggest that genetic algorithms may offer advantages in convergence time compared with other mainstream optimisation strategies [59,60], particularly in noiseless settings. The specific algorithm implemented in this study was adapted from that which was used in Refs. [17,37,58], in order to assess minimalist engineering protocols within power–law LR models. The fitness function for the specific algorithm employed within this study is

(6) $$\begin{array}{c} x\left(f_{1}^{N}\left(\tau \right),\tau \right)=A\;e^{\left(a(\right|f_{1}^{N}{\left(\tau \right)|}^{2}-1))}, \end{array}$$

where A=100 and τ is the time at which maximum excitation transfer fidelity is attained. The parameter *a* is a constant set to 10, consistent with previous studies. The time τ (in units of $J_{0}^{-1}$) is within the time interval of $t\cdot J_{0}\in \left[0,\frac{N\pi }{4}\right]$, where $\frac{N\pi }{4J_{0}}$ is the speed limit for NN chains [18]. The algorithm commences via an initialisation of a population of randomly generated individuals of long-range fixed-alpha chains, as described by Equation (1), where the bulk of the chain spacing is homogeneously spaced, and provides the referential spacing set to unity. Depending on the number of sites that are being optimised, the algorithm initialises a random configuration beginning from the outer sites, maintaining mirror symmetry. Therefore, if there is only one site being optimised from the beginning of the chain, then one position and local magnetic field is randomly generated and then subjected to the fitness evaluation via the fitness function Equation (6). Note that in order to vary the couplings of one (or more) end sites, whilst maintaining the chosen LR power–law exponent, the dimensionless position of that site is varied, to change all the relevant couplings via Equation (2). As mirror symmetry is enforced throughout the mutation process, this assesses also the identical position and magnetic-field profile on the opposite side of the chain. An example chain with four optimised energies and spacings is depicted in Figure 2, with the optimisations mirrored to the opposite end of the homogeneous bulk. For this study, the site positions were constrained to an inter-site distance $d_{i}$ within the range $d_{i}\in \left[0.5,5.0\right]$, while the local magnetic fields were restricted to $B_{i}\in \left[0,10J_{0}\right]$. These bounds were selected to remain within experimentally plausible parameter regimes, where site-resolved on-site energy modulations can be engineered using magnetic-field gradients [61], external harmonic potentials [62], or specific tuning of optical superlattice potentials [63]. We also enforce the bounds of the positions’ variability to ensure that no edge couplings exceed the characteristic energy (bulk coupling) of the system by more than an order of magnitude through adjacent sites becoming arbitrarily close to one another.

Once the fitnesses have been evaluated and recorded, a crossover function exchanges 50% of the genetic encoding of each of the parents to create the offspring. This process allows for the traits which might allow for the highest-fidelity transfer to be passed on to the next generation of prospective solutions. The initial genomes are defined as uniform power–law LR chains with each genome in the population pool then mutated five times to generate a random initial population. During each mutation, a position within the genome is selected at random (within the initial bounds) and a random integer less than or equal to the maximum mutation rate is generated. This value is then either added to or subtracted from the selected position, with the maximum mutation rate decreasing with generation number, as specified in Table 1. Consequently, if only a single site is being optimised from the beginning of the chain, only that position, and therefore its corresponding on-site energy, is randomly mutated before the resulting genome is evaluated using the fitness function given in Equation (6). The iteration over these individuals across a sufficiently large number of generations and populations allows the optimisation to converge towards high-performing solutions, with convergence assessed once the stopping criterion of a maximum number of generations is reached. The fine tuning of the mutation rate, particularly one that decreases over generations, is advantageous for finding more precise regions of local optima.

## 5. Results

Here, we present the results from the optimisation approach described by the previous section. For fair comparison across ranges of *N* and α, we set $J_{0}$ =1 as the characteristic energy scale for the dynamical analysis.

### 5.1. Distinct Modes of Transfer

**Figure 3:**
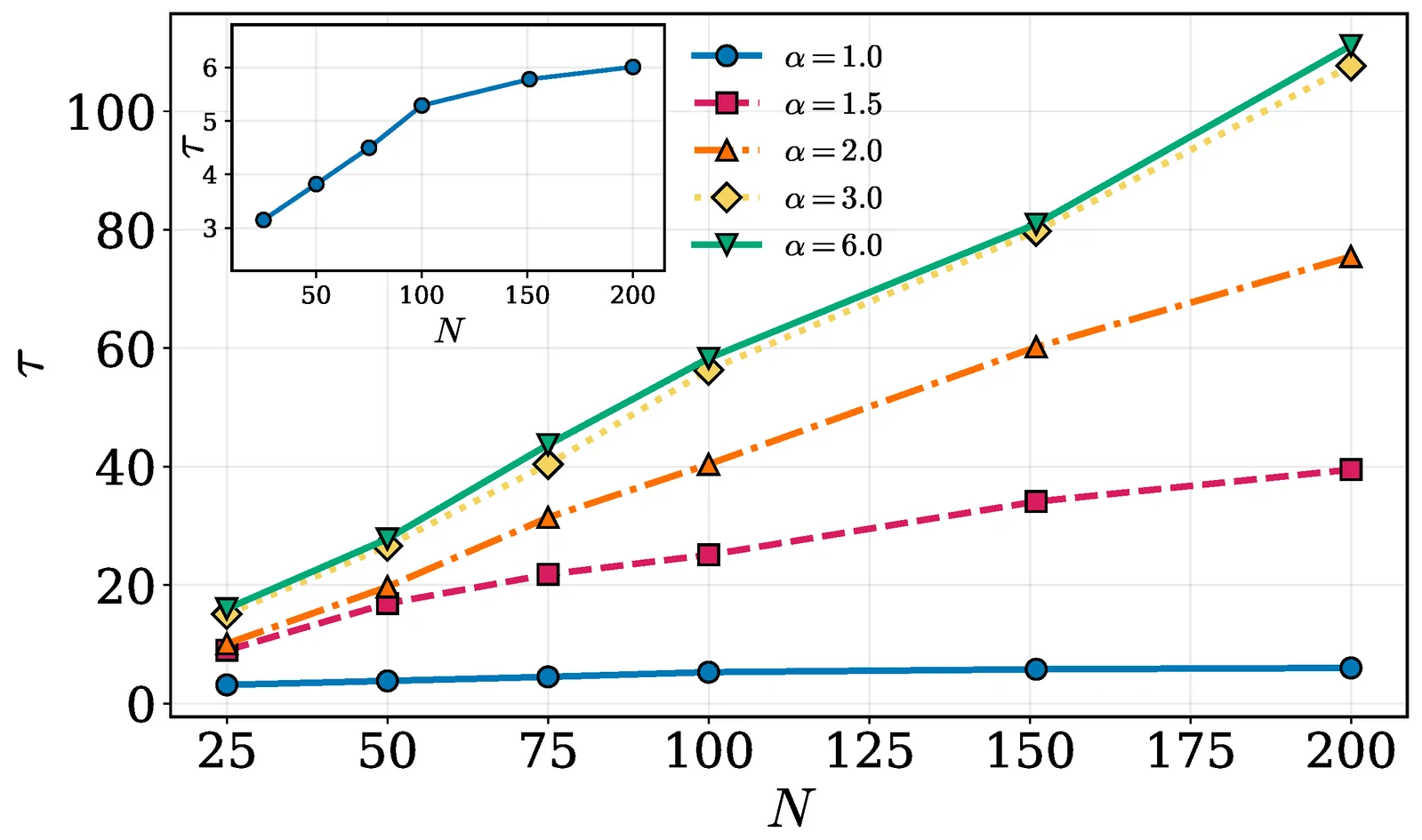
Time at which the fidelity is maximal τ plotted as a function of the total number of sites *N*, across increasing strengths of the power–law exponent α. Inset presents a zoomed-in window of the α=1 chains, highlighting the distinguishability of the individual data points.

**Figure 4:**
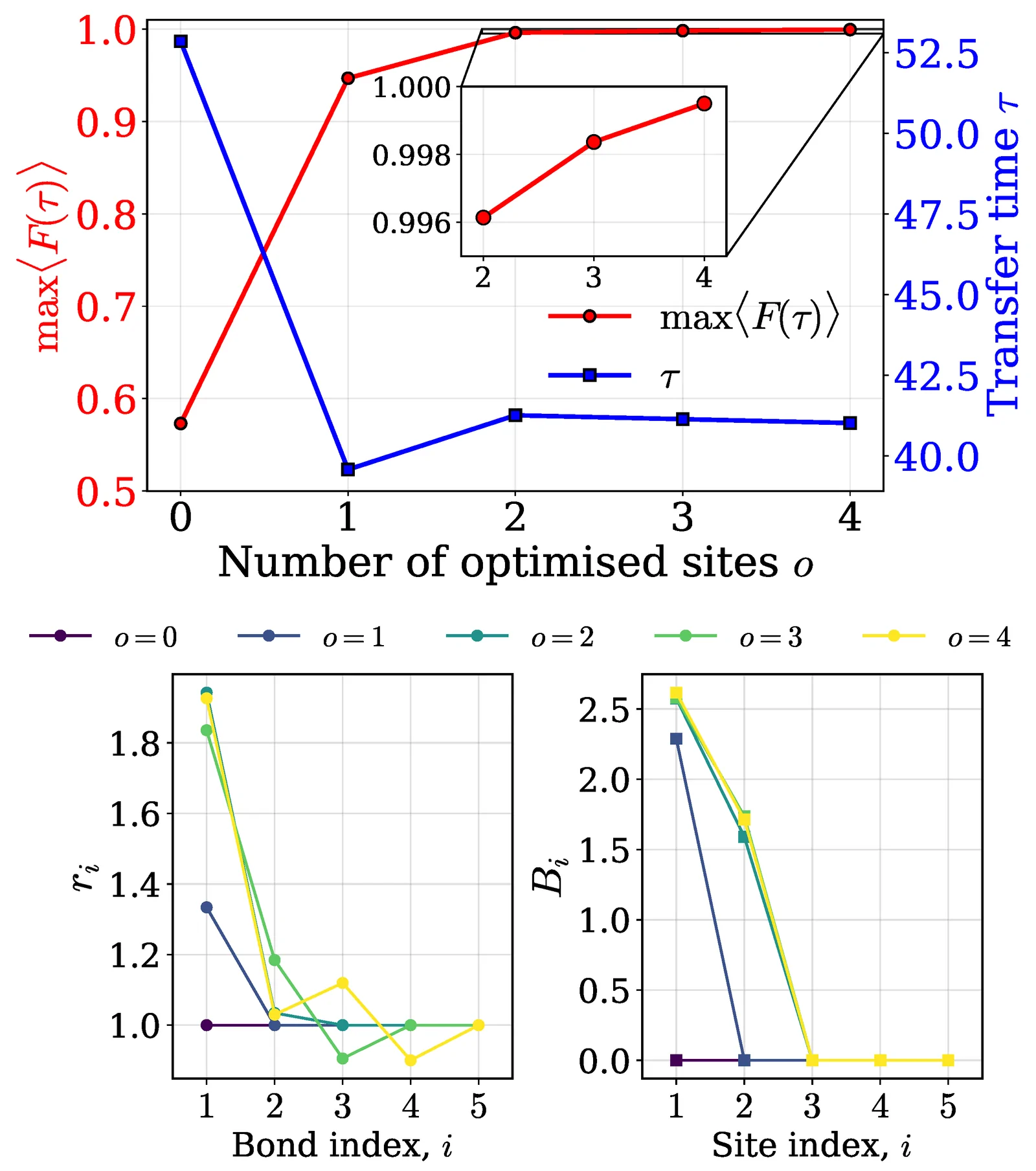
Top panel shows, for a fixed N=100 site long-range (α=2) chain, how increasing the number of optimised boundary sites *o*, from 0 to 4 leads to near-perfect ( 99.99%) average fidelity and fast transfer time, eventually approaching a saturation regime. Bottom panel illustrates the corresponding optimised spatial profiles of the spacing between sites and local magnetic fields across the lattice: the bulk remains homogeneous with uniform bond strength and zero field modulation, while non-trivial structure develops only near the boundaries.

**Figure A1:**
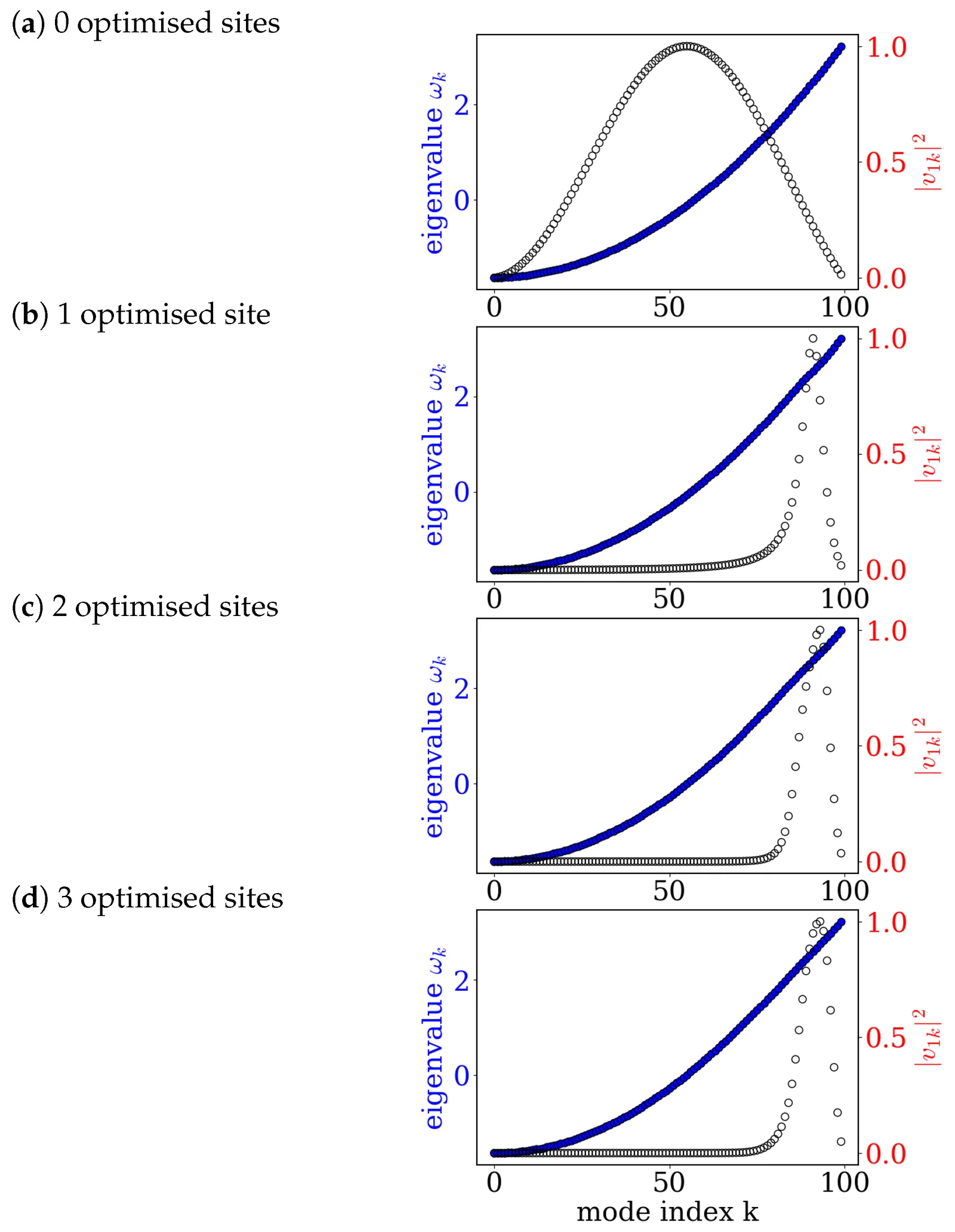
The increasing-ordered spectrum $\omega_{k}$ (blue circles) and initial wavepacket in *k*-space $v_{1k}^{2}$ (white circles) across different optimisation configurations for the α=2.0 power–law LR chain and N=100. The differences in the appearance of the initial wavepacket in the *k*-space between (**d**) and 4 optimised sites is indistinguishable, therefore 4 is omitted.

Table A1 summarises the QPST performance achieved using the optimised boundary-coupling schemes for system sizes $N=10^{1}-10^{2}$. For the great majority of systems, a maximum average fidelity of 0.999 is reached. Figure 3 plots τ as a function of *N* across the varied α-systems, demonstrating the scaling of the time at which the average fidelity achieves its maximal value for the optimised solutions. This is sublinear for α≤1.5. Figure 4 shows how increasing the number of optimised sites for a fixed chain length and interaction exponent (α=2.0) leads to saturation of both the maximum fidelity and the corresponding time τ at which it is attained. The bottom panel illustrates how modest modifications to the site positions and on-site energies are sufficient to achieve the highest fidelity for a given number of optimised boundary sites *o*. Correspondingly, Figure A1 (see Appendix A) presents how the increase in number of optimised sites yields the appearance of more participatory eigenmodes concentrated within a linear region near the top of the energy-ordered spectrum.

**Figure 5:**
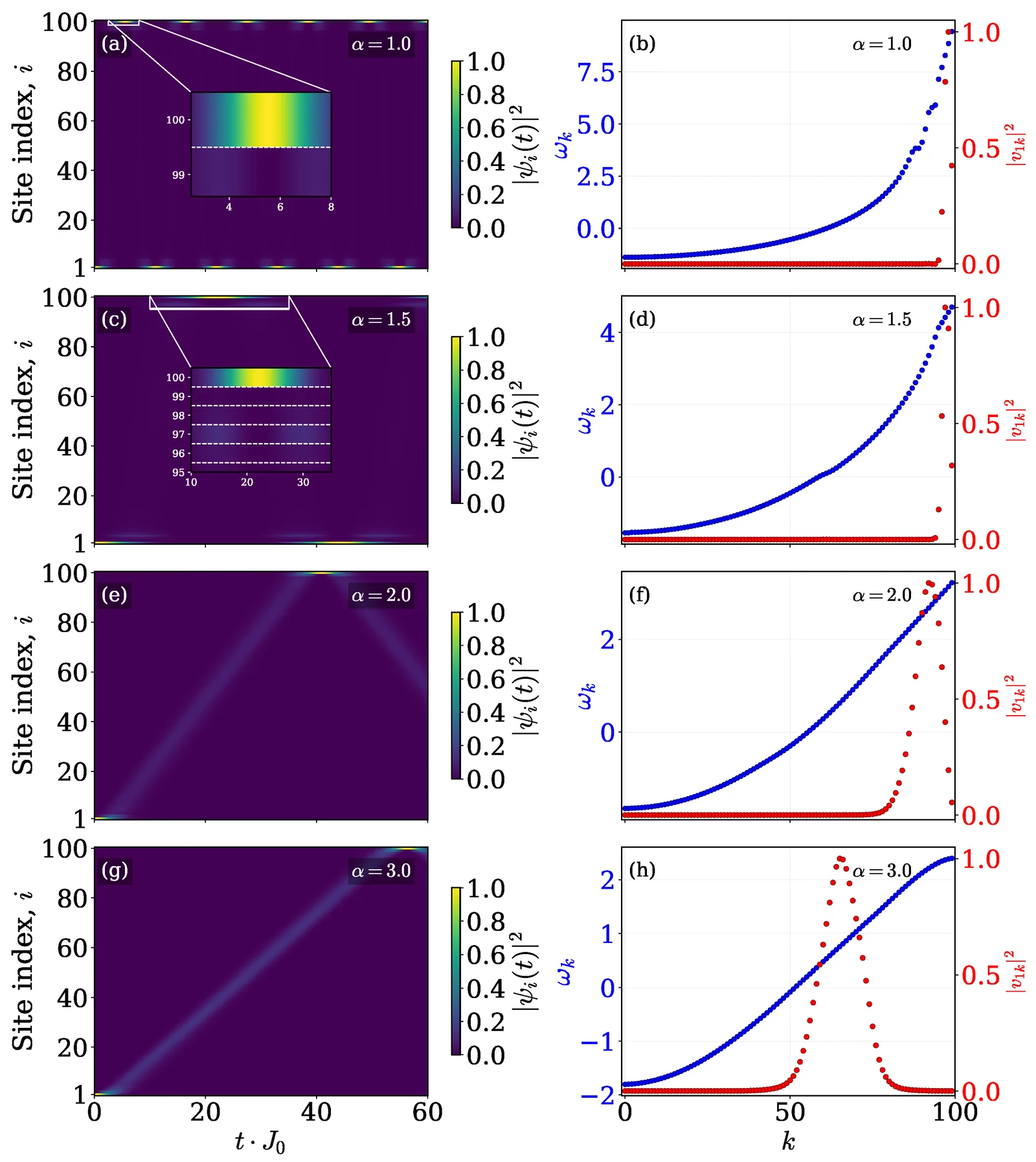
Distinct transfer dynamics associated with optimised chains for the selected sequences of α values (left column), alongside the localisation of the initial-state distribution in *k*-space over the ordered spectrum (right column) of the corresponding varied-α, N=100-site chain. Panels (**a**,**c**,**e**,**g**) show the site-resolved transfer dynamics, for α=1.0,1.5,2.0, and 3.0, respectively, while panels (**b**,**d**,**f**,**h**) show the corresponding ordered eigenvalue spectra, $\omega_{k}$ (blue circles), together with the normalised initial-state weights, $|v_{1k}{|}^{2}$ (red circles).

Figure 5 shows the dynamics, spectra, and initial-state distributions in *k*-space for different values of α. The left column illustrates the spatial spread and localisation of the excitation as a function of time, while the right column shows the corresponding initial-state distribution and energy spectrum of the optimised solutions. A table detailing each configuration shown in Figure 5 is provided in Table A2 (see Appendix B). For α=3.0, the spectral concentration of the initial-state distribution and dynamics are highly consistent with both α=6.0 (hence its omission) and previous ballistic transfer schemes [15]. Namely, this distribution localises, with many participatory-modes, in a mostly linear segment of the ordered spectrum. This close agreement suggests that the transfer mechanism is somewhat ballistic in nature. To examine this, we consider the symmetry properties of the Hamiltonian’s energy eigenvectors. Ballistic wavepacket transfer schemes exploit the mirror symmetry inherent to Hamiltonians that support high-fidelity quantum state transfer, for which the first and last components of the $k^{\mathrm{th}}$ eigenvector satisfy $v_{1k}={\left(-1\right)}^{k}v_{Nk}$ [64]. Consequently, Equation (5) can be rewritten as

(7) $$\begin{array}{c} f_{1}^{N}\left(t\right)=\sum_{k=1}^{N}v_{1k}^{2}e^{-i\left(\omega_{k}t-k\pi \right)}\;. \end{array}$$

This expression admits a natural interpretation as a wavepacket whose spectral components are weighted by $v_{1k}^{2}$ and evolve according to the dispersion relation $\omega_{k}$. Following Refs. [15,17,39], the ballistic wavepacket approach to achieve high-fidelity quantum state transfer is to engineer the spectral weight ($v_{1k}^{2}$) so that it is concentrated within a region where the ordered spectrum ($\omega_{k}$) is approximately linear. In this regime, all significant spectral components propagate with nearly identical phase velocities $\omega_{k}/k$, suppressing dispersion and enabling ballistic excitation transfer.

For 1≤α≤1.5, in order to converge to the most optimal solutions, the total optimisation time window was progressively shortened: at difference with α≥1.6, higher-fidelity solutions were found at significantly shorter transfer times, compared to other α solutions, within the prescribed bounds on the positions and magnetic fields. The resultant dynamics of these much faster solutions exhibit a markedly different regime of state transfer. This behaviour can be characterised as few-mode coherent oscillations between the end sites. This has some similarities to part of the nearest-neighbour system cases, where an effective Hamiltonian can emerge with only a few eigenstates entering the dynamics of Equation (5) [8,21,22]. In these cases, however, these few-states dynamics did not lead to fast transfer. Figure 5 shows that the N=100, α=1.0 transfer is effectively mediated by just the highest four equally spaced energy eigenstates, with all others playing a negligible role. See Appendix C for further discussion of this transfer mechanism underlying the α=1.0 solutions.

**Figure 6:**
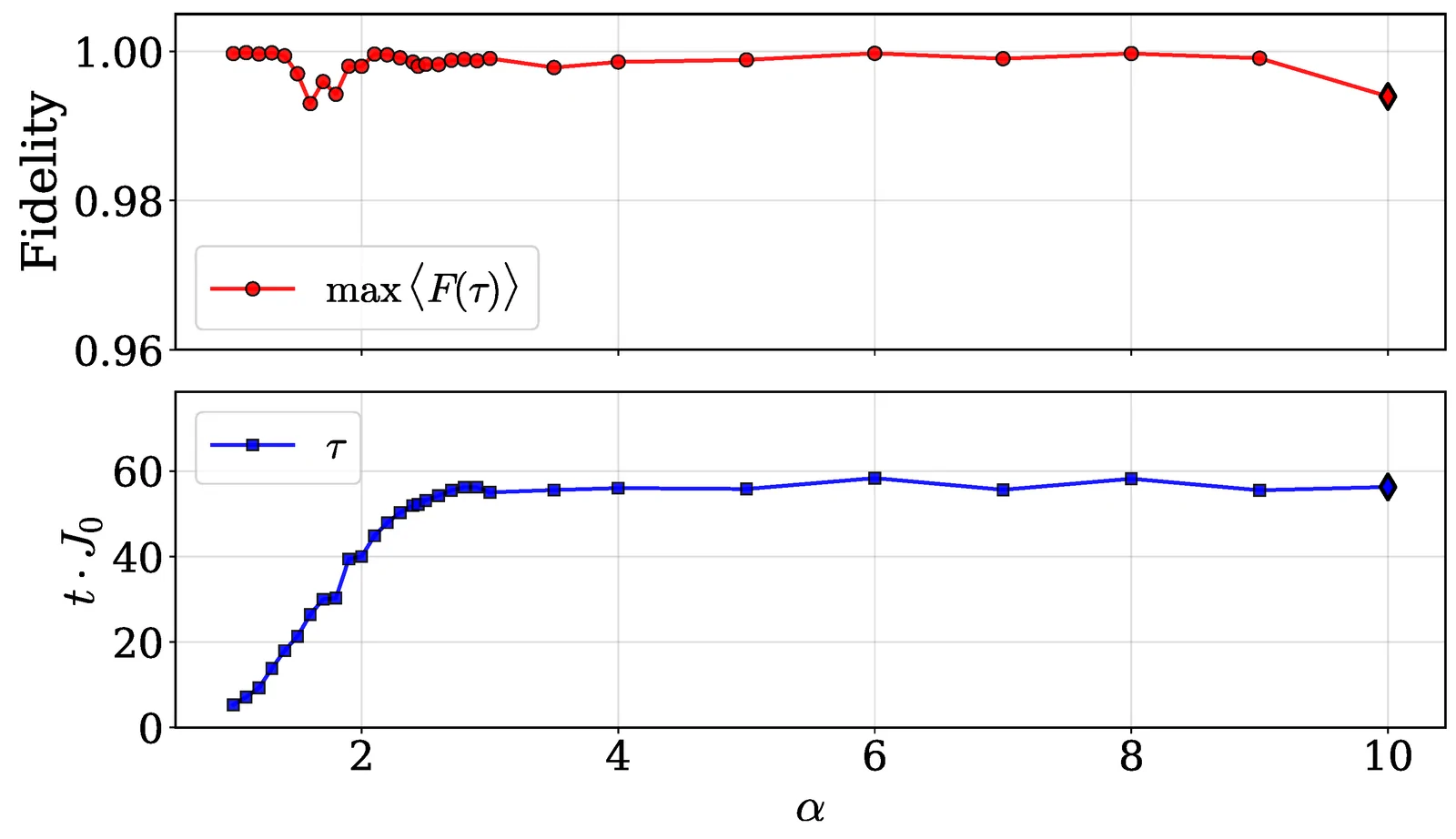
A plot of the maximal average fidelities and time taken for N=100 optimised chains for various α-values between 1 and 10. Diamonds at the (right) end of the plots represent an overlap with the exact values of average fidelity, and time at which the fidelity is attained, for the exclusively nearest-neighbour case [15].

For α=1.5, then on to α=2.0, there is a growth in the number of energy eigenstates contributing to the transfer dynamics, with a wavepacket in k-space emerging, growing in width with increasing α. Figure 3 and Figure 6, demonstrate that, for fixed *N*, the optimal transfer time τ is shortest for the lowest value of α=1.0. The time grows with increasing α, as more energy eigenstates contribute and the wavepacket in k-space emerges and widens, up to approximately α=3, when these growths effectively saturate. Notably, as shown in the upper panel of Figure 6, the maximum fidelity remains always above 0.993, with a small region for 1.0≤α≤2.0 in which the maximum fidelity decreases slightly. This is accompanied by a discontinuous increase in the time τ at which the maximum fidelity is attained. We suggest that this region is indicative of the transitional regime between the few-mode coherent oscillation mechanism and ballistic wavepacket propagation.

**Figure 7:**
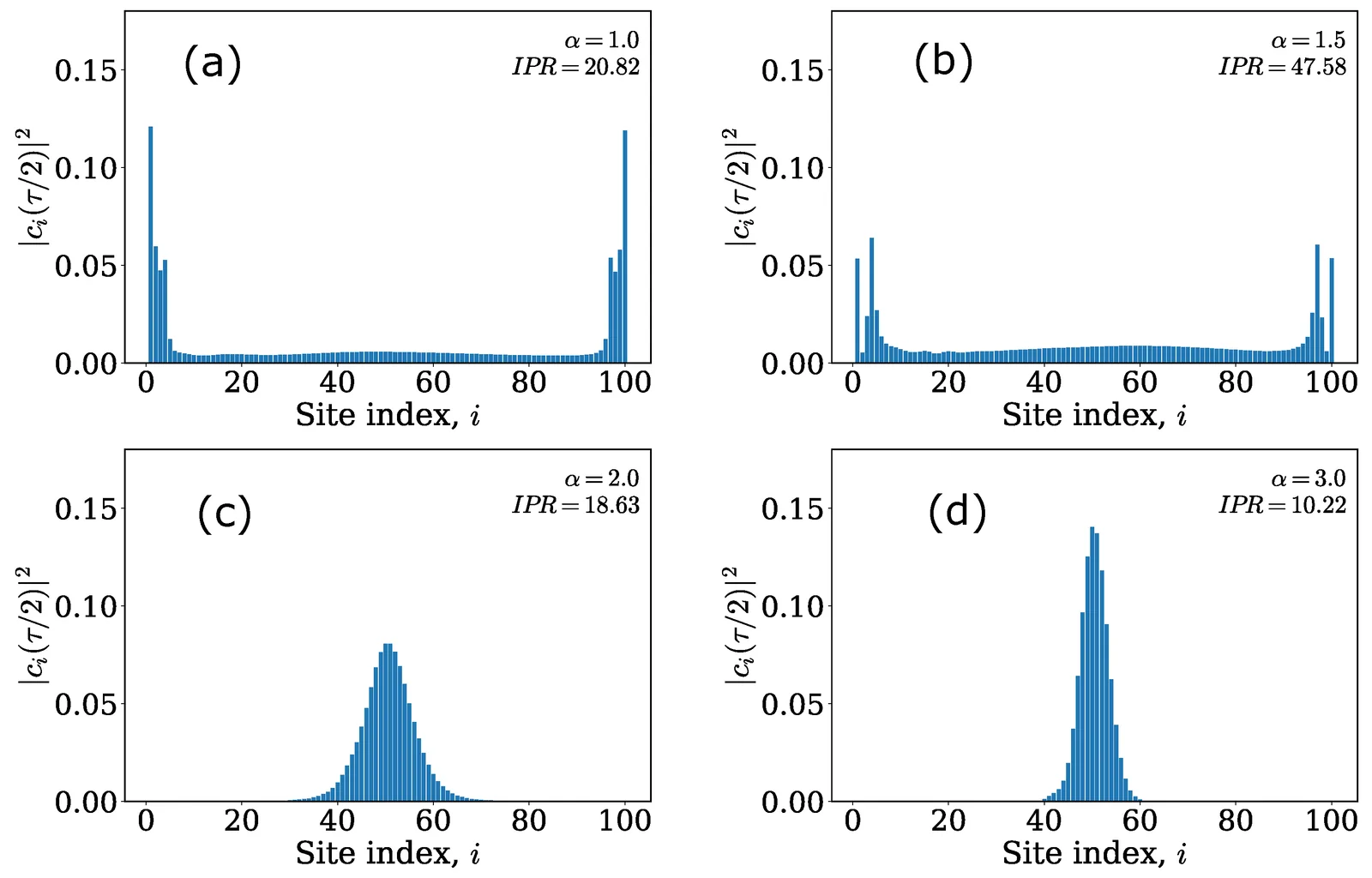
2D snapshots of the transfer fidelity of the excitation to each site for the optimised *N* = 100−site chains at $t=\tau_{max}/2$ for α=1.0, 1.5, 2.0, and 3.0 in panels (**a**–**d**), respectively. The *IPR* (Equation (8)) value for each snapshot is provided to give a quantitative measure for the wavepacket spread.

Figure 7 shows a snapshot of the transfer fidelity to each site at half the transfer time, τ, for four optimised chains. There is a marked difference between the snapshots for α≤1.5, showing the probability distribution bunched at the chain sides, and α≥2, displaying a bell curve at the centre of the chain. Writing the state of the system as $|\Psi \left(t\right)\rangle =\sum_{i=1}^{N}c_{i}\left(t\right)\;|i\rangle ,$ where |i〉 denotes the basis state with the excitation localised at site *i*, the inverse participation ratio (*IPR*) is defined as [34,65]

(8) $$\mathrm{IPR}=\frac{\sum_{i}{\left|c_{i}\right|}^{2}}{\sum_{i}{\left|c_{i}\right|}^{4}}\;,$$

which provides a quantitative measure of the delocalisation of the wavepacket. The *IPR* values shown within Figure 7 are also evaluated at τ/2, when it should reach its maximal value IPR∈[1,N]. The IPR values and distribution snapshots are very similar for α=3.0 and 6.0, hence the latter is omitted. The α=1.5 chain has a significantly higher *IPR* than the rest and a more visually spread-out distribution, indicating a shift in transfer dynamics. This was also accompanied by a change in the adjustments made to the sites, with neighbouring end sites having much larger differences in on-site energies as well as being pulled further away from the bulk for α<2.0. For α=1.0, the *IPR* is significantly lower than with α=1.5, despite the wavepacket having appreciable components across almost every site in the chain. The probability distribution nevertheless remains more strongly localised at the edges, with lower amplitudes across the intermediate sites. The characteristic bell curve observed for the α=2.0 chain is here completely lost. The excitation instead remains largely concentrated on the edge sites, indicating a distinct form of transfer for chains with higher connectivity.

Our results in Figure 6 show that, when normalising the transfer dynamics by the largest coupling in the bulk, the transfer occurs at times much faster than the speed limit in the nearest-neighbour case [18]. However, often is the case for the results presented, particularly for α<3, that the maximal coupling $J_{max}$ for the optimised solutions actually goes beyond (though never beyond an order of magnitude larger than) the coupling of the bulk, as we allow for the site spacing at the mirror–symmetric edges to come closer than the bulk site spacing. While we expect that normalising the transfer time by $J_{\max}$, which can exceed $J_{0}$ for some of the optimised solutions, leads to quantitative shifts in τ, the qualitative trend of decreasing transfer time with decreasing α remains unchanged. We still normalise with respect to the bulk coupling for the sake of comparison, as this is the only coupling value that is consistent across all α-values and chain lengths. We also observe that, for the α=1 solutions, the magnetic fields required to induce the few-mode coherent oscillations underlying the exceptionally fast QPST are often well approximated by the largest eigenvalue of the corresponding unperturbed Coulomb-interaction Hamiltonian with zero local field differences. This observation suggests that the field strength required to realise this distinct transfer mechanism may exhibit only weak system-size dependence. This weak system-size dependence is consistent with the modest growth of the largest eigenvalue with *N* for the α=1 Coulomb interaction, implying that substantially larger systems require only a modest increase in the corresponding boundary-field scale. We therefore conjecture that, despite the increasing spectral radius of the unperturbed Coulomb Hamiltonian with system size, the required magnetic fields on the sender and receiver sites grow only modestly and remain experimentally tractable for substantially larger systems. Table 2 compares our proposed protocol with previously reported long-range engineering protocols under natural dynamics for high-fidelity QST.

### 5.2. Robustness

**Figure 8:**
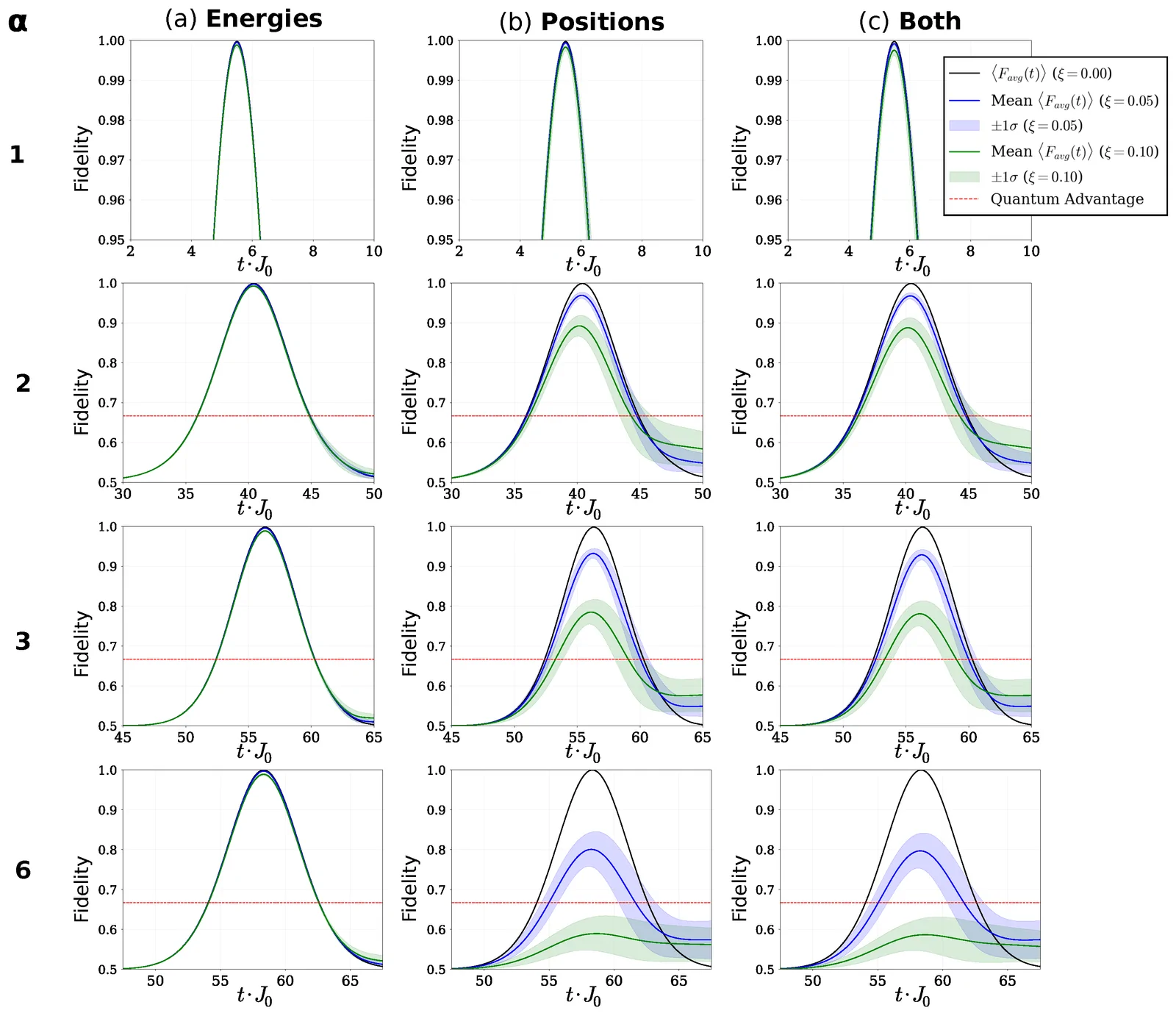
Plots detailing the effect of introducing random disorder into the on-site energies and positions of optimised N=100 chains for the four interaction strengths of interest. The columns show the effects of perturbing the site energies alone (**a**), the site positions alone (**b**), and both simultaneously (**c**). The α=1 panels are zoomed-in as the fidelity scale is much more concentrated due to disorder having significantly less effect as other α-values.

Furthering the goal of experimental feasibility, we have investigated the effect of random asymmetric disorder (i.e., disorder that does not preserve mirror symmetry) on both the position profile and on-site energies within the optimised LR chain solutions. See Appendix B for an analysis of the temporal robustness of the unperturbed solutions corresponding to those presented in Table A2. The on-site energies are modified as

(9) $$\varepsilon_{i}^{\mathrm{eff}}=\varepsilon_{i}+\xi \;a_{i},\;i=1,\ldots ,N,$$

$$a_{i}∼U\left[-0.5,0.5\right],$$

where ξ denotes the disorder strength (e.g., ξ=0.1 corresponds to a 10% fluctuation amplitude). Similarly, the inter-site distances entering are perturbed according to

(10) $$r_{i}^{\mathrm{eff}}=r_{i}\left(1+\xi b_{i}\right),\;i=1,\ldots ,N-1,$$

$$b_{i}∼U\left[-0.5,0.5\right],$$

where $a_{i}$ and $b_{i}$ are independent random variables and U is the uniform sampling. For the results presented within Figure 8, the effective local magnetic field/on-site energies (Figure 8a), and positions (Figure 8b) are averaged over 1000 samples, to collect the mean fidelity values and standard error. Figure 8c combines the two. The noise simulations presented a significant robustness in the mean transfer fidelity ≥96% up to a disorder error strength of 5% (ξ=0.05) for positional noise, and up to 10% (ξ=0.10) for on-site energy noise. It is important to note that asymmetric errors in the range of 5–10% are relatively large in the context of state-of-the-art trapped-ion and optical-lattice platforms. As the site positioning on quantum hardware can be achieved with high spatial precision [66,67], the error model considered in Figure 8 should be interpreted as a deliberately conservative scenario, intended to investigate the robustness of the worst-case against imperfections in the positions of the sites. Therefore, the results presented in Figure 8 represent a strict benchmark for the stability of the protocol under experimentally pessimistic conditions.

Figure 8 shows that across all considered interaction strengths, random disorder in the on-site energies has very little effect on fidelity. As displayed in Figure A1, the on-site energy of the first site is responsible for positioning the initial wavepacket in k-space, whereas the energies of subsequent sites subtly shift the spectrum of the chain. It therefore follows that random disorder of the order $0.1J_{0}$ would only have a significant effect on the first site. In the cases of α=1.0 and 2.0, the first on-site energy was $>1J_{0}$ for the optimised configurations tested, meaning the disorder strength is smaller in comparison. Also, as shown in Figure 5, the ordered spectrum for α=3.0 has a significantly larger linear section, suggesting that small changes to the magnetic-field strengths would not move the wavepacket out of the linear section. This is similarly the case for α=6.0.

The effect of random disorder in the positions, however, tells a much more interesting story. As α increases, so too does the effect of disorder to the point that a 10% disorder for α=6.0 loses quantum advantage. However, a 5% disorder strength for α=3.0 retains >0.9 average fidelity to ±1σ, implying a reasonable level of robustness to relatively large positional disorder. For α=1.0, there was almost no change to average fidelity, even at 10% disorder strength. Even when simultaneously applying disorder to both positions and on-site energies, the α=1.0 configuration retained >0.99 average fidelity to ±1σ at 10% disorder strength. Furthermore, for sufficiently large systems (N∼75–200), we found that specific boundary-engineered solutions exhibit a stability under addition of a few sites to the bulk while maintaining nearly identical average fidelity values. This highlights the robustness and adaptability of these solutions, which may be exploited when exact system sizes cannot be realised. While the robustness to variations in the on-site energies is consistent with that observed for the other interaction strengths, the pronounced robustness to positional disorder at α=1.0 is particularly striking. Not only does the α=1.0 regime enable significantly faster transfer, but it also appears to exhibit an intrinsically greater tolerance to positional imperfections. It is possible that this robustness to changes in site positions is due to the higher interaction strength; each site has a stronger connection to surrounding sites and can better average out perturbations. Also, it is possible that this is due to the limited number of active eigenmodes displayed in Figure 5. Since the majority of the modes are dynamically inactive, perturbations applied to the vast majority of bulk sites would have little effect, resulting in the observed robustness. However, further robustness analyses are beyond the scope of this study. The timescales of the state-transfer dynamics considered here are well within the experimentally demonstrated coherence times $T_{2}$ of several relevant quantum platforms, including trapped ions, semiconductor spin qubits, and NV centres; representative values are summarised in Table 3. Thus, such decoherence is expected to have only a limited effect on the state-transfer dynamics considered here. Future work, applying our generic, flexible model to specific experimental hardware, could include decoherence relevant for that specific hardware.

## 6. Conclusions and Outlook

We have presented distinct transfer mechanisms within optimised power–law, long-range-interacting spin-$\frac{1}{2}$ systems. While very-high fidelity solutions are found for all power–laws, fast transfer is particularly associated with power exponents 1≤α≤1.5, also showing favourable sublinear scaling with chain length. These results are found using a minimalist engineering approach yielding a realistic pathway to experimental implementation and subsequent benchmarking. Furthermore, robustness analyses present, for systems with higher connectivity than an effective nearest-neighbour system, a marked improvement in robustness to static disorder/parameter imprecision. This result further underscores the implementability and potential witnessing of the distinct modes of transfer in real hardware.

## Figures and Tables

**Table 1:** Genetic algorithm parameters used for the optimisation of spin-chain configurations for high-fidelity state transfer.

Parameter	Value
Selection method	Roulette-wheel selection
Crossover method	Uniform crossover
Initial mutation rate	20%
Final mutation rate	1%
Generations	100
Genomes per generation	4096
Time divisions per unit time	100
Stopping criterion	Maximum generations reached
Reported value	Best genome found within individual runs

**Table 2:** Comparison of different LR engineering protocols under natural dynamics for chains of length N=100. The table lists the interaction type, engineering approach, peak fidelity, and transfer time in units of $J_{0}^{-1}$. All power–law interactions considered here follow α=3.0, except for the Extended-XY model, for which α=2.7.

Protocol	Interaction	Engineering	〈F(τ)〉	$\tau \cdot J_{0}$
Our protocol	XX, power–law	Boundary positions + fields	0.998	56.3
PST NNN [13]	XX, NNN	Full-system engineering	1.000	$10^{3}$
Ballistic NNN [17]	XX, NNN	Boundary couplings + one field	0.999	68.9
Double-hole [27]	XX, power–law	2 sites removed	0.999	$10^{6}$
Extended-XY [32]	XY, power–law	Anisotropy parameters	0.779	120

**Table 3:** Comparison of different hardware in terms of operation time and coherence. $J_{0}$ corresponds to the typical energy scale reported in the cited literature, and τ is the estimated time required for high-fidelity transfer for N=50 ($\tau =3.82/J_{0}$, $26.6/J_{0}$, $27.8/J_{0}$ for α=1,3, and 6 respectively), which is within the dephasing time $T_{2}$.

Hardware	$J_{0}/\hbar$	τ	$T_{2}$
NV Centre (α=3) [40,68]	1 MHz	27 μs	∼100 ms
Cold Atoms (α=6) [41,48]	10 MHz	2.8 μs	>1 ms
Ions (α=1) [42]	1 kHz	3.8 ms	∼seconds
Superconductors (α=3) [44,69]	100 MHz	0.27 μs	100 μs

**Figure A2:**
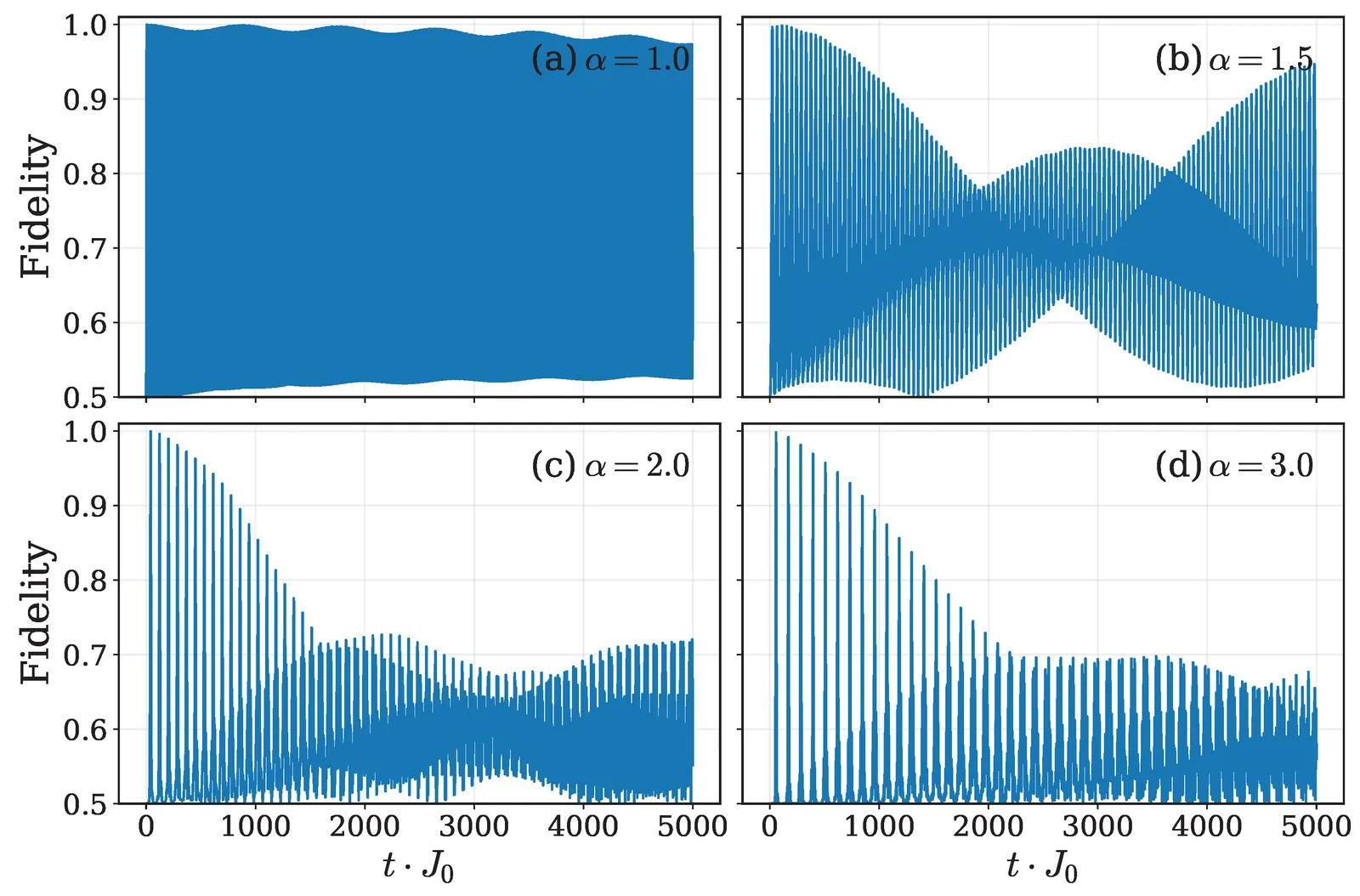
Average QST fidelity as a function of $t\cdot J_{0}$ for *N* = 100 using the optimised boundary positions and fields shown in Table A2 for the different values of the power–law exponent α.

**Figure A3:**
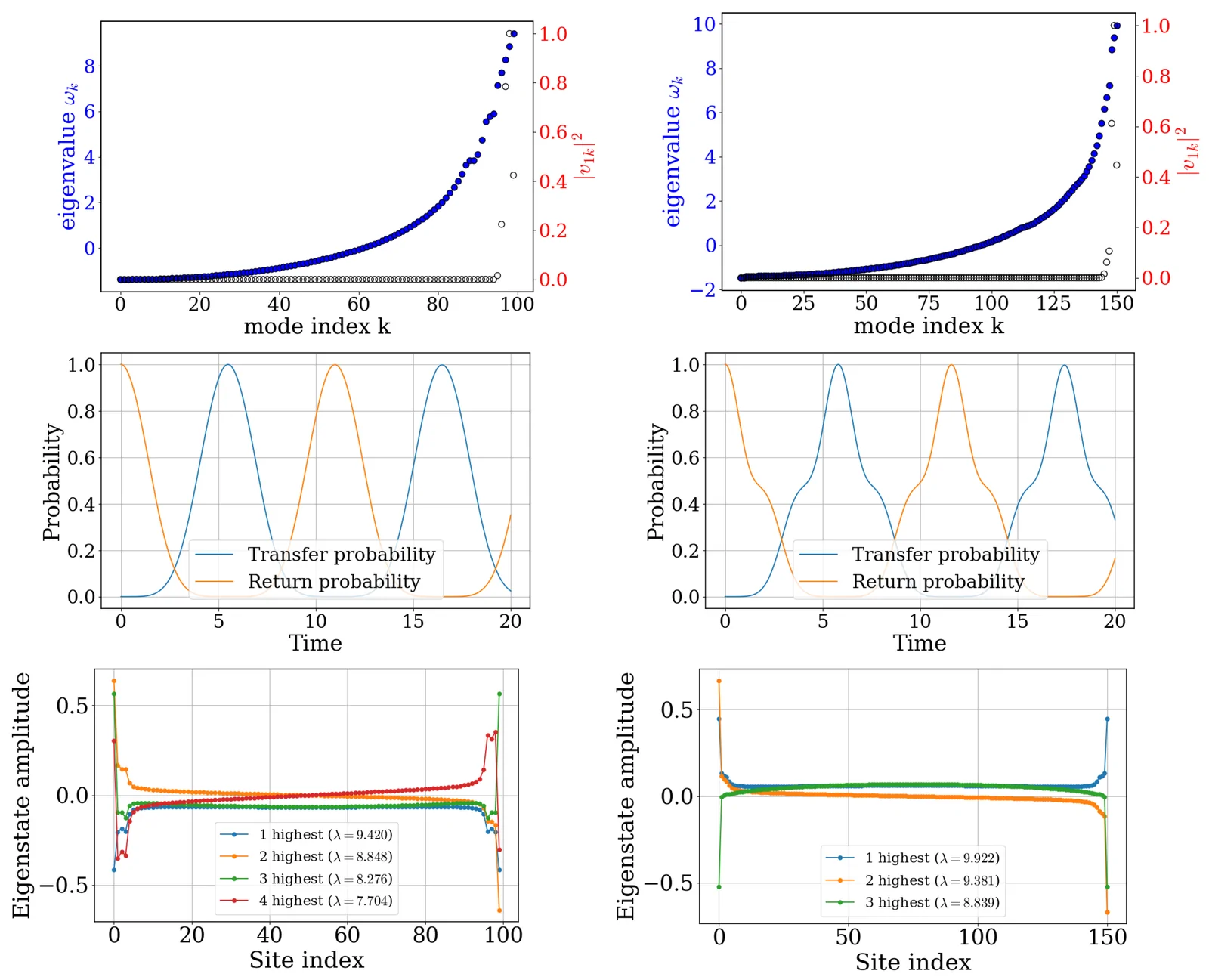
Initial-state localisation, transfer-return dynamics, $|f_{1}^{N}{\left(t\right)|}^{2}$ and $|f_{1}^{1}{\left(t\right)|}^{2}$ respectively (Equation (5)), and the site-basis amplitudes of the high-energy participatory eigenstates for α=1, shown for N=100 (left column) and N=151 (right column).

## Data Availability

Due to group policy the data supporting the findings of this work is available upon reasonable request.

## Abbreviations

The following abbreviations are used in this manuscript:

NN	nearest-neighbour
NNN	next-nearest-neighbour
LR	long-range
QST	quantum state transfer
PST	perfect state transfer
QPST	quasiperfect state transfer

## Appendix A

Table A1 summarises the maximum average fidelity and corresponding arrival time for the different chain lengths and power–law exponents considered. The maximum average fidelity remains close to unity across all configurations, demonstrating that high-fidelity state transfer can be maintained over a broad range of *N* and α. In contrast, the corresponding arrival time τ increases systematically with both chain length and α. The increase is particularly pronounced for larger α, reflecting the progressively shorter-ranged nature of the interactions and the resulting slower propagation of quantum information through the chain. Figure A1 shows the corresponding eigenvalue spectrum and initial wavepacket weights in *k*-space as the number of optimised boundary sites is increased. While optimisation substantially modifies the spectral structure and wavepacket distribution initially, the differences become progressively less pronounced as more boundary sites are optimised. In particular, the four-site configuration is visually indistinguishable from the three-site case in *k*-space and is therefore omitted for clarity.

**Table A1:** Maximum average fidelity and corresponding arrival time $\left(\max\langle F\left(t\right)\rangle ,\tau_{\max}\right)$ for several chain lengths *N* and power–law exponents α.

〈F(τ)〉;τ					
N	α=1.0	α=1.5	α=2.0	α=3.0	α=6.0
25	0.999;3.15	0.999;8.99	0.999;10.1	0.999;15.1	0.999;16.0
50	0.999;3.82	0.999;16.9	0.999;19.7	0.999;26.6	0.999;27.8
75	0.999;4.50	0.999;21.8	0.999;31.4	0.998;40.4	0.999;43.7
100	0.999;5.50	0.999;25.0	0.999;40.4	0.998;56.3	0.999;58.3
151	0.999;5.78	0.999;34.1	0.999;60.2	0.999;79.76	0.999;80.96
200	0.999;6.01	0.997;39.5	0.998;75.48	0.998;107.7	0.999;111.1

## Appendix B

The optimised boundary parameters corresponding to the solutions presented in Figure 5 are listed in Table A2. The results show a clear dependence of the optimal boundary configuration on the interaction exponent α. For α=1.0 and 1.5, the optimised parameters exhibit larger deviations from the homogeneous bulk, whereas for increasing α the optimised parameters progressively approach those of the unmodified chain. In particular, several of the boundary fields become negligible or vanish entirely for α≥1.5, while the boundary spacings likewise become closer to unity. This indicates that increasingly short-ranged interactions require less pronounced boundary engineering to achieve the optimised transfer observed in Figure 5 and Figure 6. Figure A2 shows the decay in fidelity of the optimised solutions presented in Table A2. The peak fidelity of the α=1 solution exhibits only minimal oscillatory behaviour and remains above 0.99 throughout the time window shown. For the other values of α, the fidelity gradually decreases but remains above the classical communication threshold of 2/3 [51] well into the regime of $t\cdot J_{0}∼10^{3}$. This persistence of high-fidelity transfer suggests that the minimally engineered solutions can retain a quantum advantage over extended evolution times, providing additional flexibility for experimental implementations and repeated transfer attempts.

**Table A2:** Optimised boundary parameters for mostly homogeneous N=100 long-range chains with four modified boundary sites. The bulk couplings are uniform, with only the boundary spacings $d_{i}=r_{i,i+1}$ and local fields $B_{i}$ optimised under various power–law exponent strengths.

Parameter	α=1.0	α=1.5	α=2.0	α=3.0
$r_{1,2}$	3.970	4.780	1.926	1.429
$r_{2,3}$	0.660	1.120	1.029	1.227
$r_{3,4}$	1.736	0.696	1.119	1.105
$r_{4,5}$	0.500	0.741	0.900	1.047
$B_{1}$	8.644	4.450	2.616	0.769
$B_{2}$	5.770	0.000	1.713	0.537
$B_{3}$	5.051	0.000	0.000	0.235
$B_{4}$	5.503	1.690	0.000	0.113

## Appendix C

For α=1.0, the dynamics exhibit few-mode coherent oscillations between the end sites. Figure 5 and Figure A3 demonstrate that for N=100 and N=151, the dynamics are dominated by the highest four and three energy eigenstates, respectively. These dynamics are a little more involved than the simplest form of coherent oscillations involving just two energy eigenstates. Furthermore, this latter simple scenario usually involves two near-degenerate eigenvalues, which, since the transfer time relates to the reciprocal of the energy level separation, inevitably provides only very long transfer times.

The advantage of involving just three or four eigenstates, with equally spaced eigenvalues, is that the transfer time, which again relates to the reciprocal of the separation, is much shorter. As can be seen from the site-basis decompositions of the participating eigenstates also shown in the bottom panels of Figure A3, an analogy can be drawn with three- and four-site NN PST chains. With both three and four states, the highest energy eigenstate has even symmetry about the chain centre. The symmetry then oscillates between odd and even as the energy of the participating state decreases. In both examples, a specific superposition of the few participating states corresponds to the excitation completely localised at site i=1. At the mirror time, when the odd participating eigenstates have acquired a phase factor of (−1) relative to the even eigenstates, this superposition corresponds to the mirror of the initial state, with the excitation thus completely localised at site i=N. This mirroring works in an analogous manner to mirroring in NN PST chains [2,3].

As already stated, the mirror or transfer time relates to the reciprocal of the energy level separation $t=\frac{\pi }{\Delta \omega_{i,i+1}}$, so the transfer can be much faster than oscillations due to two almost degenerate states. Clearly, the solutions presented here do not support the mirroring of any multi-qubit initial state, because in each example the whole spectrum is not equally spaced and with oscillating symmetry. Only the mirroring of an arbitrary single-qubit quantum state injected at site i=1 is to be expected, because that is the problem that the optimisation approach was set up to deliver.
